# Binding of calcium and magnesium to human cardiac troponin C

**DOI:** 10.1016/j.jbc.2021.100350

**Published:** 2021-02-03

**Authors:** Kaveh Rayani, Justin Seffernick, Alison Yueh Li, Jonathan P. Davis, Anne Marie Spuches, Filip Van Petegem, R. John Solaro, Steffen Lindert, Glen F. Tibbits

**Affiliations:** 1 Molecular Cardiac Physiology Group, Simon Fraser University, Burnaby, British Columbia, Canada; 2 Department of Chemistry and Biochemistry, Ohio State University, Columbus, Ohio, USA; 3 Department of Biochemistry and Molecular Biology, The University of British Columbia, Vancouver, British Columbia, Canada; 4 Department of Physiology and Cell Biology, The Ohio State University, Columbus, Ohio, USA; 5 Department of Chemistry, East Carolina University, 300 Science and Technology Building, Greenville, North Carolina, USA; 6 Department of Physiology and Biophysics and the Center for Cardiovascular Research, College of Medicine, University of Illinois at Chicago, Chicago, Illinois, USA; 7 Department of Molecular Biology and Biochemistry, Simon Fraser University, Burnaby, British Columbia, Canada; 8 Cardiac Group, BC Children's Hospital Research Institute, Vancouver, British Columbia, Canada

**Keywords:** contractility, myofilament, ITC, calorimetry, MD simulation, thermodynamic integration, molecular dynamics, CaM, calmodulin, cTn, cardiac troponin, cTnC, cardiac troponin C, cTnI, cardiac troponin I, cTnT, cardiac troponin T, FF, fast-flow, ITC, isothermal titration calorimetry, PDB, Protein Data Bank, sTnC, skeletal muscle troponin TnC, TF, thin filament, TI, thermodynamic integration

## Abstract

Cardiac muscle thin filaments are composed of actin, tropomyosin, and troponin that change conformation in response to Ca^2+^ binding, triggering muscle contraction. Human cardiac troponin C (cTnC) is the Ca^2+^-sensing component of the thin filament. It contains structural sites (III/IV) that bind both Ca^2+^ and Mg^2+^ and a regulatory site (II) that has been thought to bind only Ca^2+^. Binding of Ca^2+^ at this site initiates a series of conformational changes that culminate in force production. However, the mechanisms that underpin the regulation of binding at site II remain unclear. Here, we have quantified the interaction between site II and Ca^2+^/Mg^2+^ through isothermal titration calorimetry and thermodynamic integration simulations. Direct and competitive binding titrations with WT N-terminal cTnC and full-length cTnC indicate that physiologically relevant concentrations of both Ca^2+^/Mg^2+^ interacted with the same locus. Moreover, the D67A/D73A N-terminal cTnC construct in which two coordinating residues within site II were removed was found to have significantly reduced affinity for both cations. In addition, 1 mM Mg^2+^ caused a 1.4-fold lower affinity for Ca^2+^. These experiments strongly suggest that cytosolic-free Mg^2+^ occupies a significant population of the available site II. Interaction of Mg^2+^ with site II of cTnC likely has important functional consequences for the heart both at baseline as well as in diseased states that decrease or increase the availability of Mg^2+^, such as secondary hyperparathyroidism or ischemia, respectively.

Cardiac troponin (cTn) is a heterotrimeric complex that includes components for Ca^2+^ binding (cardiac troponin C [cTnC]), inhibition of contraction (cardiac troponin I [cTnI]), and tropomyosin binding (cardiac troponin T [cTnT]) (1). Ca^2+^ binding to site II of cTnC is the precursor to a series of structural perturbations in the thin filament (TF) that culminate in a force-generating reaction between the actin filament and myosin heads (1, 2, 3, 4, 5).

Human cTnC is a 161-amino acid protein composed of nine helices (N and A–H), which form four EF-hand or helix–loop–helix binding motifs (sites I–IV). Within these domains, residues in positions 1 (+x), 3 (+y), 5 (+z), 7 (−y), 9 (−x), and 12 (−z) contain oxygen atoms arranged in a pentagonal bipyramid allowing for coordination of metal cations (Figs. S1 and S2) (6, 7, 8). Skeletal muscle troponin TnC (sTnC) has four functional Ca^2+^-binding motifs (9, 10). cTnC has a similar overall structure to sTnC but a slightly different primary sequence; the insertion of a valine at residue 28, along with the substitutions D29L and D31A, has rendered site I of cTnC nonreceptive to Ca^2+^ binding (11, 12).

Ca^2+^ binding to sites III and IV in the C domain of cTnC occurs with high affinity (∼10^7^ M^−1^) (∼10× higher than the N domain) and a slow exchange rate (∼100× slower than binding to the N domain) (13, 14, 15). Given the abundance of contractile filaments throughout cardiomyocytes, sites III/IV of cTnC buffer ∼80% of the 100 to 200 μM [Ca^2+^]_in_ at resting concentrations of free Ca^2+^ (∼100 nM) (16). At resting free cytosolic Ca^2+^ concentrations, sites III and IV are usually saturated with Ca^2+^ (16). Mg^2+^ also binds at sites III and IV but with lower affinity (*K*_A_ ∼ 10^4^ M^−1^) (4). However, the cytosolic concentration of Mg^2+^ allows this cation to compete with and reduce the binding of Ca^2+^ to the “structural” sites (17, 18). The binding of Ca^2+^/Mg^2+^ to sites III and IV alters the structure of TnC and is a prerequisite for tethering to the rest of the TF (19, 20).

The C domain of cTnC is linked to the N domain by a linker region composed of a nine-turn α-helix (21, 22). Within the N domain (N-cTnC), Ca^2+^ binds the low-affinity (∼10^−5^ M) site II such that this site is only partially occupied at diastolic-free Ca^2+^ concentrations (∼0.1 μM) with very few sites being bound (23). The degree of occupancy is significantly higher at systolic-free Ca^2+^ concentrations (∼0.5–1.2 μM), which follow Ca^2+^-induced Ca^2+^ release (24). Ca^2+^ binding to site II provides the free energy to allow for exposure of a hydrophobic pocket, which is otherwise less favorable (25, 26). Helices B and C move away from helices N, A, and D to expose the hydrophobic cleft, with the short antiparallel β-sheet between EF-hands I and II acting as a hinge (27, 28, 29). Binding of the “switch peptide” of cTnI_147–163_ to this pocket facilitates exposure of this hydrophobic region (22, 30, 31).

A persistent and underinvestigated question is the role of cellular Mg^2+^ in the signaling of activation by cTnC. Of the total intracellular [Mg^2+^]_i_ (∼10 mM), the majority is bound to cellular components such as ATP with only ∼0.5 to 1.0 mM being freely available in the cytosol (32, 33). In conditions with diminished Mg^2+^ buffering capacity, such as ATP-depleted states, the free [Mg^2+^] can increase significantly (34, 35) prior to being extruded from the cell (36), but it could also compete with Ca^2+^ for binding to cTnC.

Increase in Mg^2+^–ATP in both skeletal and cardiac tissues decreases the Ca^2+^ sensitivity of skinned fibers (37, 38, 39). These systems contain the entirety of the TF. The binding of Ca^2+^ normally induces a conformation change, which allows for exposure of the hydrophobic core of cTnC (40). It is unlikely that Mg^2+^ would cause this structural change. Indeed, previous findings suggest that Mg^2+^ competes for binding with Ca^2+^ but does not itself cause the same conformational changes (20).

Further evidence has been obtained through fluorescence-based studies of isolated cTnC (41, 42, 43, 44, 45, 46), the Tn complex (41, 43), and reconstituted fibers (43, 47), where Mg^2+^ appears to decrease Ca^2+^ sensitivity. In isolated TnC, the *K*_A_ of the low-affinity sites (III/IV) for Ca^2+^ and Mg^2+^ was measured to be on the order of 10^6^ and 10^2^ M^−1^, respectively (42).

Interaction of Ca^2+^/Mg^2+^ with sites III/IV results in a large change in enthalpy (ΔH), in contrast to changes resulting from site I/II binding, which are small by comparison. Detection of heat changes associated with the interactions of metal ions and proteins is both challenging and a highly technique-dependent method, such that small changes may be deemed negligible (48, 49, 50). Experiments used to study this system decades ago were limited by the technology of the time. In contrast, modern isothermal titration calorimetry (ITC) is a sensitive method that can be used to define the thermodynamic parameters of binding without the use of labeling methods that could interfere. While there is a place for use of fluorophores to investigate cTnC function (51, 52, 53), even conservative substitutions (*e.g.*, sTnC F29W) have been shown to modify Ca^2+^-binding properties (54). Modern ITC allows for the study of single-binding sites within isolated protein domains and can be used to detect heat changes as small as 0.1 μcal (55, 56, 57, 58).

We have used ITC to explore the binding of Mg^2+^ and Ca^2+^ to site II at the level of N-cTnC and full-length cTnC. Competitive binding to the N domain and mutations in the site II caused a reduction in apparent affinity, indicating interaction of both cations with the same locus in the protein. In addition, a double mutant removing two of the coordinating residues within the EF-hand of site II was used to investigate the impact on Ca^2+^ and Mg^2+^ binding to this site. In full-length cTnC, Mg^2+^ competed with and reduced Ca^2+^ binding to all three sites. These findings further corroborate and expand upon what has been shown by a few laboratories, but the findings are largely ignored by most; the role of Mg^2+^ in modulating the Ca^2+^ sensitivity of force production in cardiomyocytes is one that merits further discussion (40, 51, 52, 53).

## Results

The ratio of the ligand to titrant in the single-binding site condition (as given by the stoichiometry—N) is a measure of the functional moles of protein and was approximately 1.00 in all the N-cTnC titrations (Table S2). Given the method of concentration determination, the number of binding sites, cooperativity, and the variable binding strength of each titrants, the N cannot be used in the same way for the full-length cTnC experiments (Table S3). Therefore, the values presented can be compared between conditions, but care should be taken when comparing these to other systems, such as the cTn complex or the reconstituted thin filaments (59, 60). Ease of manipulation of the N-cTnC/cTnC system contrasts with those that include the cTn/TF. Thus, the binding parameters measured here may not translate in absolute term when cTnC is incorporated into a more complex system; these limitations are further explored in the Discussion section.

### Ca^2+^ and Mg^2+^ binding to apo-state N-cTnC

**Figure 1:**
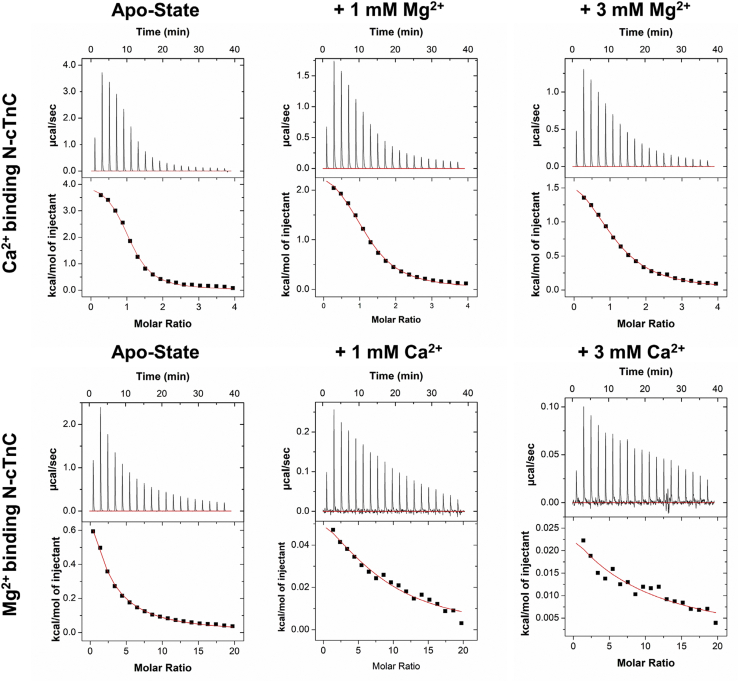
**Representative isotherms of Ca**^**2+**^**and Mg**^**2+**^**binding to apo-state and preincubated N-cTnC.** All titrations were carried out into 200 μM WT human N-cTnC in 50 mM Hepes at pH 7.2, 150 mM KCl, and 2 mM EDTA. The *top row* shows the titration of 4 mM Ca^2+^ into apo-state N-cTnC, followed by the same titration into 1 and 3 mM Mg^2+^ preincubated N-cTnC. The *bottom row* shows the titration of 20 Mg^2+^ into apo-state N-cTnC, followed by 1 and 3 mM Ca^2+^-preincubated N-cTnC. In the last injections, Mg^2+^ in the Ca^2+^-preincubation conditions, the heat changes approached the detection limits of the instrument. Thermograms were fit to a “single-binding site” model using Origin 7 MicroCal2000 ITC software package. N-cTnC, cardiac troponin C with N domain.

The interaction of N-cTnC with either Ca^2+^ or Mg^2+^ was found to be associated with a positive ΔH, so the interaction is driven by entropy (Fig. 1) consistent with previously published data (61, 62, 63, 64). Given the characteristic of the heat signals observed in the site II–containing system of N-cTnC, the endothermic component of the multiple binding site system (full-length cTnC) can be attributed to binding at this site within the N domain.

**Figure 2:**
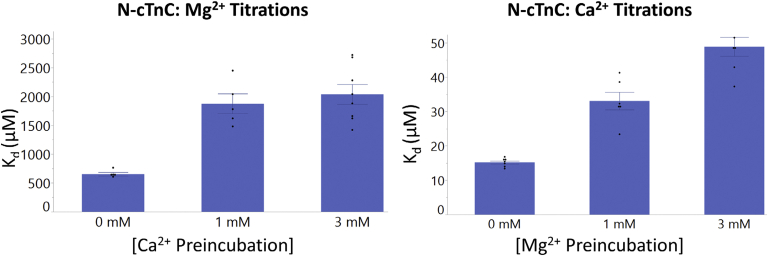
**Binding of Ca**^**2+**^**and Mg**^**2+**^**to apo-state and preincubated N-cTnC.***Left panel*, the affinity of site II for Mg^2+^ is compared in the apo-state and with Ca^2+^ preincubation in human N-cTnC. *Right panel*, the affinity of site II for Ca^2+^ is compared in the apo-state and with Mg^2+^ preincubation in N-cTnC. Statistical differences were assessed through ANOVA followed by Tukey's post hoc test. Ca^2+^ titrations were not significantly different. Titration of Mg^2+^ into N-cTnC preincubated with 1 and 3 mM Ca^2+^ was statistically indifferent, but both significantly differed from the apo-state titration (*p* < 0.0001). N-cTnC, cardiac troponin C with N domain.

The affinity of N-cTnC for Ca^2+^ (*K*_d_ = 15.2 ± 0.5 μM) was found to be significantly greater (*p* = 0.001) and 42.9-fold different than for Mg^2+^ (*K*_d_ = 652.8 ± 28.4 μM) (Figs. 1 and 2; Table S2).

The ΔH of the Ca^2+^–N-cTnC interaction (3.82 ± 0.04 kcal ∗ mol^−1^) was significantly greater (*p* < 0.0001) than that with Mg^2+^ (2.64 ± 0.10 kcal ∗ mol^−1^), indicating a greater enthalpic cost of binding for the Ca^2+^ titration. Moreover, the entropic contribution for the Ca^2+^ titrations (T ∗ ΔS = 10.39 ± 0.03 kcal ∗ mol^−1^) was more favorable (*p* < 0.0001) than the Mg^2+^ titrations (T ∗ ΔS = 6.99 ± 0.07 kcal ∗ mol^−1^) (Table S2).

As expected, the affinity of Ca^2+^ binding to apo-state N-cTnC (*K*_d_ = 15.2 ± 0.5 μM) was found to be greater than Mg^2+^ binding at this site, or when compared with the preincubation experiments (Fig. 2 and Table S2).

### N-terminal cTnC

#### Mg^2+^ binding to Ca^2+^-preincubated N-cTnC

To investigate Mg^2+^ binding in the presence of Ca^2+^, apo-state N-cTnC was preincubated with three concentrations of Ca^2+^ (0, 1, and 3 mM), then titrated with 20 mM Mg^2+^ (Figs. 1 and 2 and Table S2). Moreover, the change in enthalpy in these conditions was lower with greater amounts of Ca^2+^ preincubated. Titration of Mg^2+^ into apo-state protein yielded a ΔH = 2.64 ± 0.10 kcal ∗ mol^−1^, lower than Ca^2+^ into apoprotein, which liberated 3.82 ± 0.04 kcal ∗ mol^−1^. Moreover, the *K*_d_ values were 1870.0 ± 171.5 and 2037.5 ± 172.2 μM for the 1 and 3 mM Ca^2+^ conditions, respectively, indicating a trend of decreasing affinity with increasing concentrations of Ca^2+^ preincubated with the protein sample and a more than two orders of magnitude lower affinity compared with the Ca^2+^ into WT condition. The reduction in affinity, ΔH, and lower ΔS associated with higher Ca^2+^ preincubation suggests that both metal cations may be binding to the same EF-hand–binding motif in site II of N-cTnC.

#### Ca^2+^ binding to Mg^2+^-preincubated N-cTnC

Apoprotein preincubated with Mg^2+^ was titrated with Ca^2+^ to assess the “apparent” affinity of the protein for Ca^2+^ when the site might be occupied with the other divalent cation. As expected, increasing the Mg^2+^ concentration significantly (*p* < 0.0001) reduced the ΔH associated with binding from 3.82 ± 0.04 kcal ∗ mol^−1^ in the apotitration to 1.73 ± 0.05 kcal ∗ mol^−1^ in the 3 Mm Mg^2+^ preincubated construct. The binding affinity changed from 15.2 ± 0.5 μM in the apo-state to 48.9 ± 2.8 μM in the 3 mM Mg^2+^-preincubated condition. The Ca^2+^ affinity was lower when comparing the apo-N-cTnC binding condition with higher concentrations of preincubated Mg^2+^ (Fig. 2 and Table S2).

**Figure 3:**
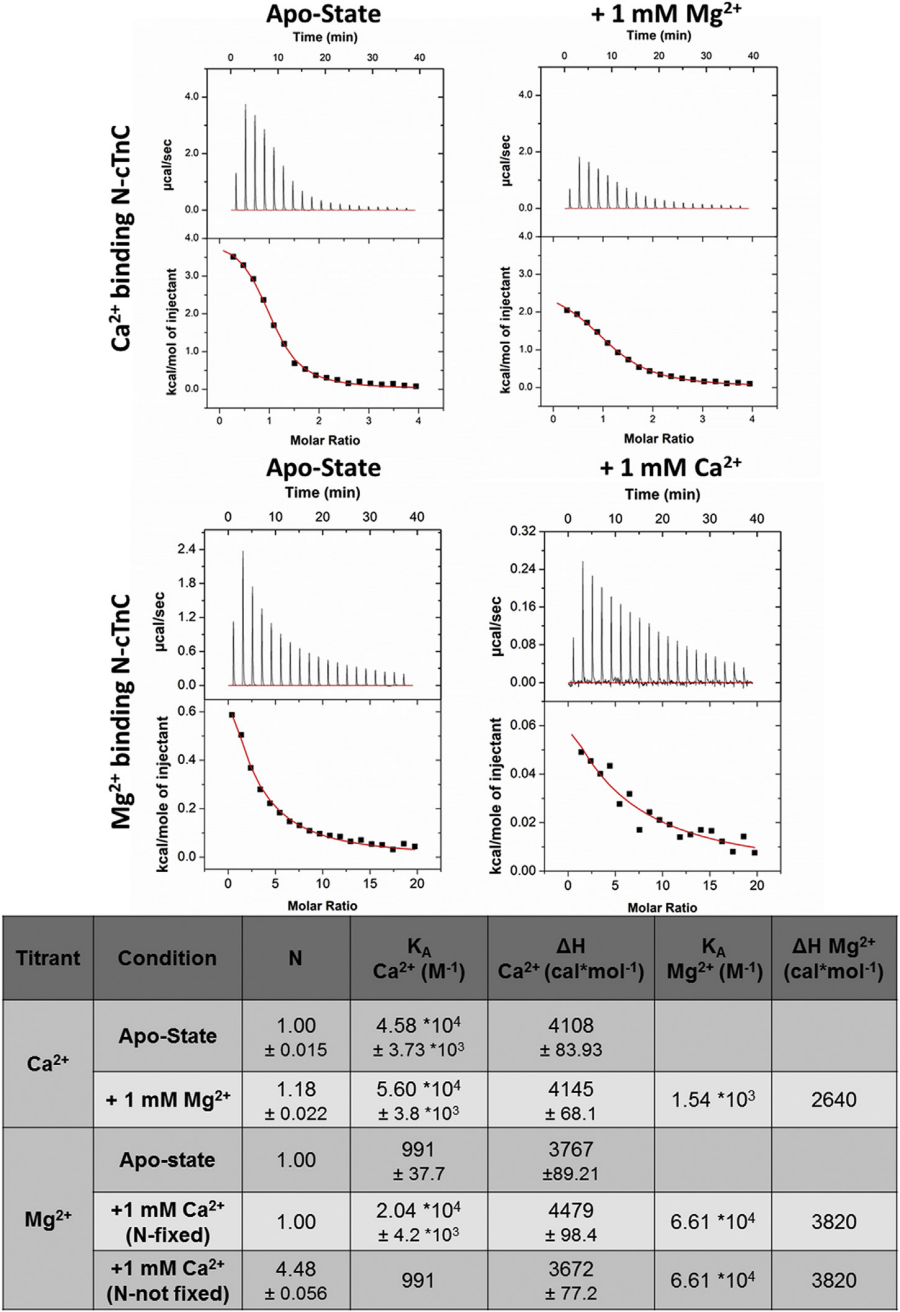
**Competition model of Ca**^**2+**^**and Mg**^**2+**^**binding to apo-state and N-cTnC preincubated with 1 mM Mg**^**2+**^**or 1 mM Ca**^**2+**^**.** All titrations were carried out into 200 μM WT human N-cTnC in 50 mM Hepes at pH 7.2, 150 mM KCl, and 2 mM EDTA. The *top two panels* show Ca^2+^ titration, and the *bottom two panels* show Mg^2+^ titrations. The *top left panel* shows the titration of 4 mM Ca^2+^ into apo-state N-cTnC, and the *top right panel* illustrates the same titration with 1 mM Mg^2+^-preincubated N-cTnC. The *bottom left panel* shows the titration of 20 mM Mg^2+^ into apo-state N-cTnC, and the *bottom right panel* illustrates the same titration with 1 mM Ca^2+^-preincubated N-cTnC. The thermograms were fit using a “competition” model using Origin 7 MicroCal2000 ITC software package, in which the concentration of the ion in the cell and an estimated *K*_A_ value is input. The values for thermodynamic parameters obtained are listed in the table described. Thermodynamic parameters without a reported error value were fixed in the model. N-cTnC, cardiac troponin C with N domain.

It is possible to directly obtain a measure of the binding of a secondary ion when a competition experiment is carried out. Figure 3 shows the binding of Ca^2+^ to N-cTnC in the apo-state and following preincubation with 1 mM Mg^2+^. This figure also shows the binding of Mg^2+^ to N-cTnC in the apo-state and following preincubation with 1 mM Ca^2+^. The values of the thermodynamic parameters obtained from this model for the apo-state titration were as follows: N = 1.00 ± 0.015 sites, *K*_A_ = 4.58 ± 0.37 × 10^4^ M^−1^, and ΔH = 4.11 ± 0.08 kcal/mol; these values are comparable to those obtained from the single-binding site model (Table S2). The parameters for the preincubation condition were as follows: N = 1.18 ± 0.02 sites, *K*_A_ = 5.60 ± 0.38 × 10^4^ M^−1^, and ΔH = 4.15 ± 0.07 kcal/mol for Ca^2+^; Mg^2+^ was estimated to bind with a *K*_A_ of 1.54 × 10^3^ M^−1^ and a ΔH of 2.64 kcal/mol; these values fall within the range obtained using the single-binding site model (Table S2). Moreover, the values obtained for the binding of Mg^2+^ to the apo-state N-cTnC and with preincubated Ca^2+^ are comparable to a single-binding site model (Fig. 3 and Table S2). The N-value obtained when applying the competitive binding model to the titration of Mg^2+^ into 1 mM Ca^2+^ preincubated N-cTnC (4.48 ± 0.06) is thought to result from a weak binding interaction and indicates a less reliable fit under the experimental conditions (Fig. 3).

#### Ca^2+^ and Mg^2+^ binding to apo-D67A/D73A N-cTnC

**Figure 4:**
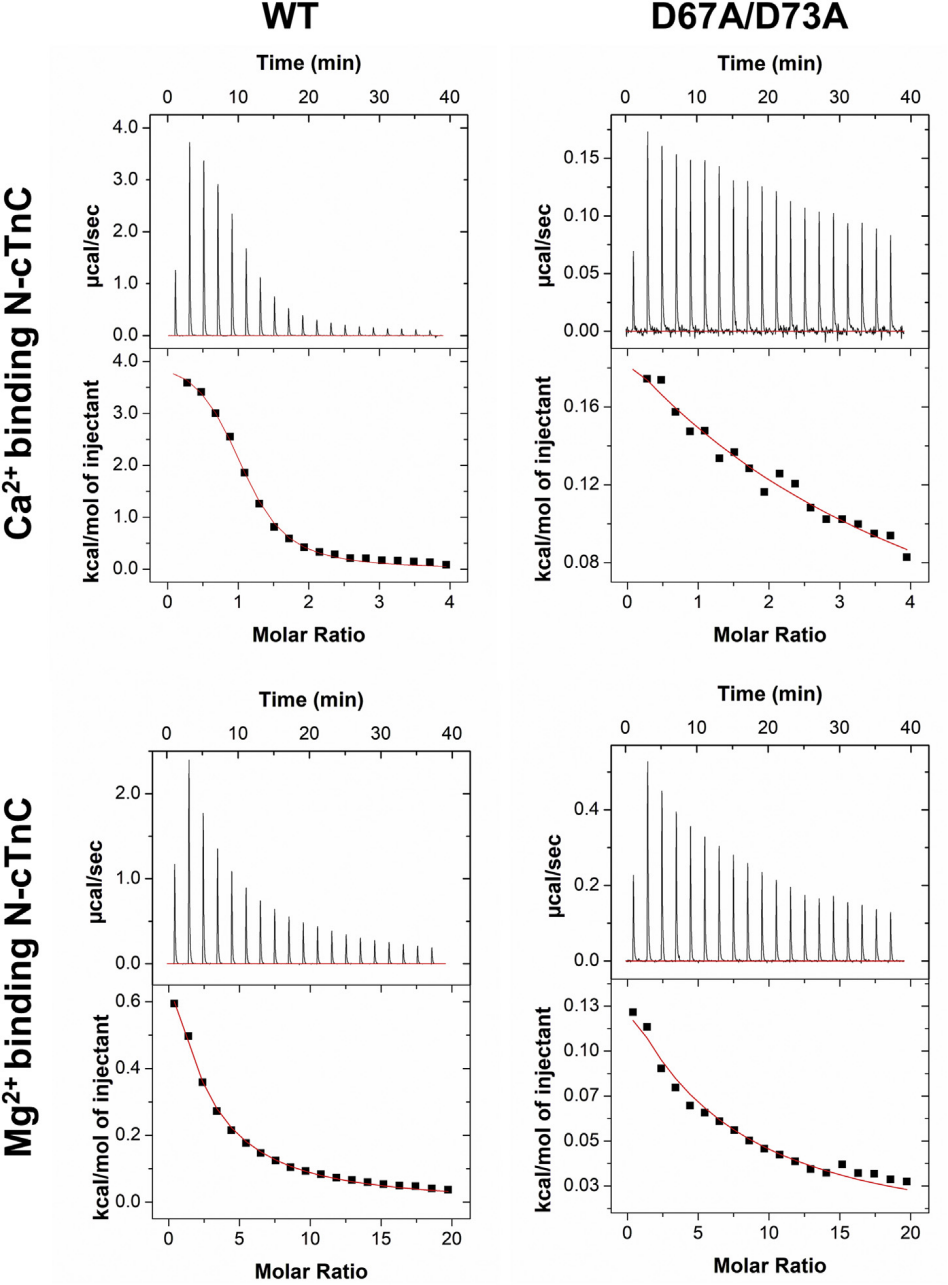
**Representative binding isotherms for binding of Ca**^**2+**^**and Mg**^**2+**^**WT and D67A/D73A N-cTnC.** All titrations were carried out into 200 μM human N-cTnC in 50 mM Hepes at pH 7.2, 150 mM KCl, and 2 mM EDTA. *Top row*, titration of 4 mM Ca^2+^ into apo-state N-cTnC, followed by the same titration into the D67A/D73A mutant. *Bottom row*, titrations of 20 mM Mg^2+^ into apo-state N-cTnC, followed by the same titration into the D67A/D73A mutant. Thermograms were fit to a “single-binding site” model using Origin 7 MicroCal2000 ITC software package. N-cTnC, cardiac troponin C with N domain.

**Figure 5:**
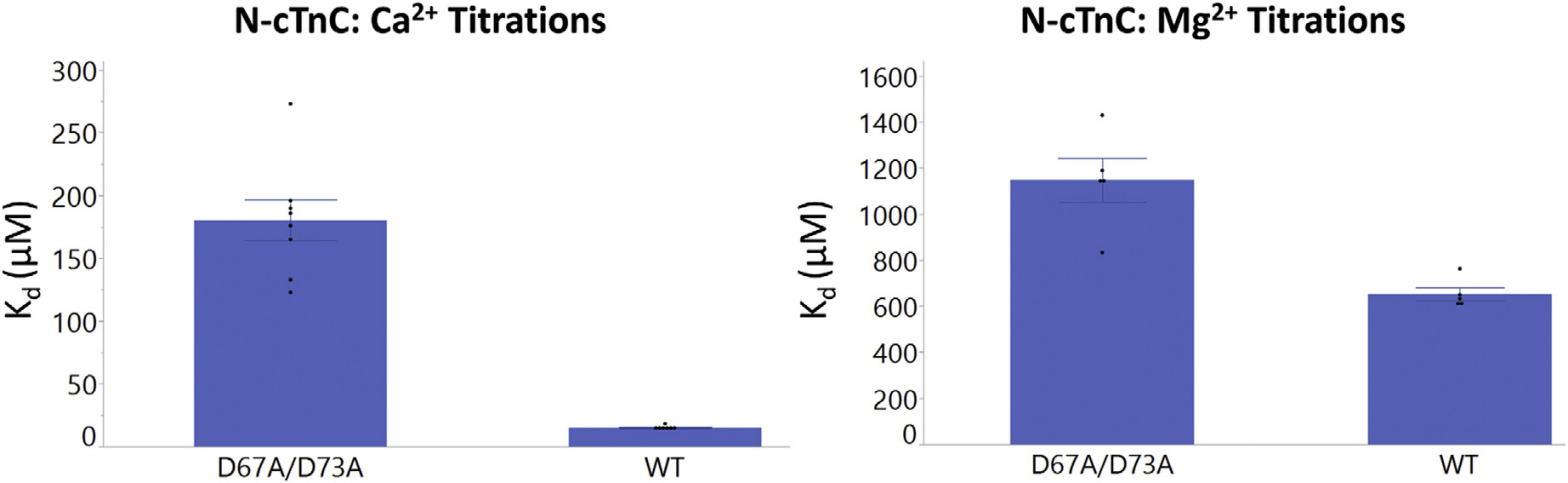
**Binding of Ca**^**2+**^**and Mg**^**2+**^**to WT and D67A/D73A N-cTnC.** The effect of the D67A/D73A on Ca^2+^ and Mg^2+^ binding is assessed. ANOVA indicated that the binding affinity for both cations to human N-cTnC was lower when comparing the mutant and the WT (*p* < 0.0001). Tukey's post hoc test indicated that all four test conditions were significantly different. The effect on Ca^2+^ binding was more pronounced (11.9-fold reduction) compared with Mg^2+^ (1.8-fold reduction), but this is reconcilable with the number of coordinating residues needed to bind Ca^2+^ (6) *versus* Mg^2+^ (5); having four available coordinating residues was expected to affect Ca^2+^ binding to a greater extent. N-cTnC, cardiac troponin C with N domain.

Point mutations (D67A and D73A) were made (Fig. S2), affecting two known Ca^2+^ coordinating residues in site II. Binding of both divalent cations was reduced by these mutations, but the *K*_d_ was lower for Ca^2+^ binding (180.3 ± 16.2 μM) compared with Mg^2+^ binding (1148.6 ± 95.0 μM) (Figs. 4 and 5 and Table S2). The double mutant caused a 11.9-fold alteration in Ca^2+^ binding, yet this difference was not found to be statistically significant (*p* = 0.88); it also altered Mg^2+^ binding 1.8-fold (*p* = 0.04); this change in *K*_d_ supports the binding of Mg^2+^ to the EF-hand of site II.

#### Ca^2+^- and Mg^2+^-binding affinities from thermodynamic integration

Thermodynamic integration (TI) was performed to calculate absolute binding affinities (with change in Gibbs free energy reported) for the ions in the following systems: Ca^2+^ to WT N-cTnC, Ca^2+^ to D67A/D73A N-cTnC, Mg^2+^ to WT N-cTnC, and Mg^2+^ to D67A/D73A N-cTnC (Table 1). The average calculated binding affinities over five independent runs were −6.9 ± 1.3, −4.5 ± 2.4, −0.6 ± 2.8, and +0.4 ± 2.3 kcal ∗ mol^−1^, respectively. The TI-determined Ca^2+^-binding affinities were in good agreement with the ITC data. While the calculated absolute Mg^2+^-binding affinities were not in perfect agreement with the ITC data, they did show that Mg^2+^ had a weaker binding affinity than Ca^2+^ for all systems (−6.57 to −4.38 kcal ∗ mol^−1^ and −6.9 to −0.6 kcal ∗ mol^−1^ for ITC and TI, respectively, for WT system and −5.12 to −4.02 kcal ∗ mol^−1^ and −4.5 to +0.4 kcal ∗ mol^−1^ for ITC and TI, respectively, for D67A/D73A system). In addition, between the Mg^2+^-binding affinities, the binding affinity was consistently weaker for the D67A/D73A mutation. The ΔΔG values comparing ΔG between WT and D67A/D73A systems were similar for ITC and TI (0.36 kcal ∗ mol^−1^ and 1.0 kcal ∗ mol^−1^, respectively).

**Table 1:** Average calculated binding affinities for Ca^2+^/Mg^2+^ interaction with site II of N-cTnC thermodynamic integration system

System	ΔG_TI_ (kcal ∗ mol^−1^)
Ca^2+^ to WT	−6.9 ± 1.3
Ca^2+^ to D67A/D73A	−4.5 ± 2.4
Mg^2+^ to WT	−0.6 ± 2.8
Mg^2+^ to D67A/D73A	+0.4 ± 2.0

Averages were calculated over five independent runs.

### Full-length cTnC

#### Ca^2+^ binding to apo-state full-length cTnC

**Figure 6:**
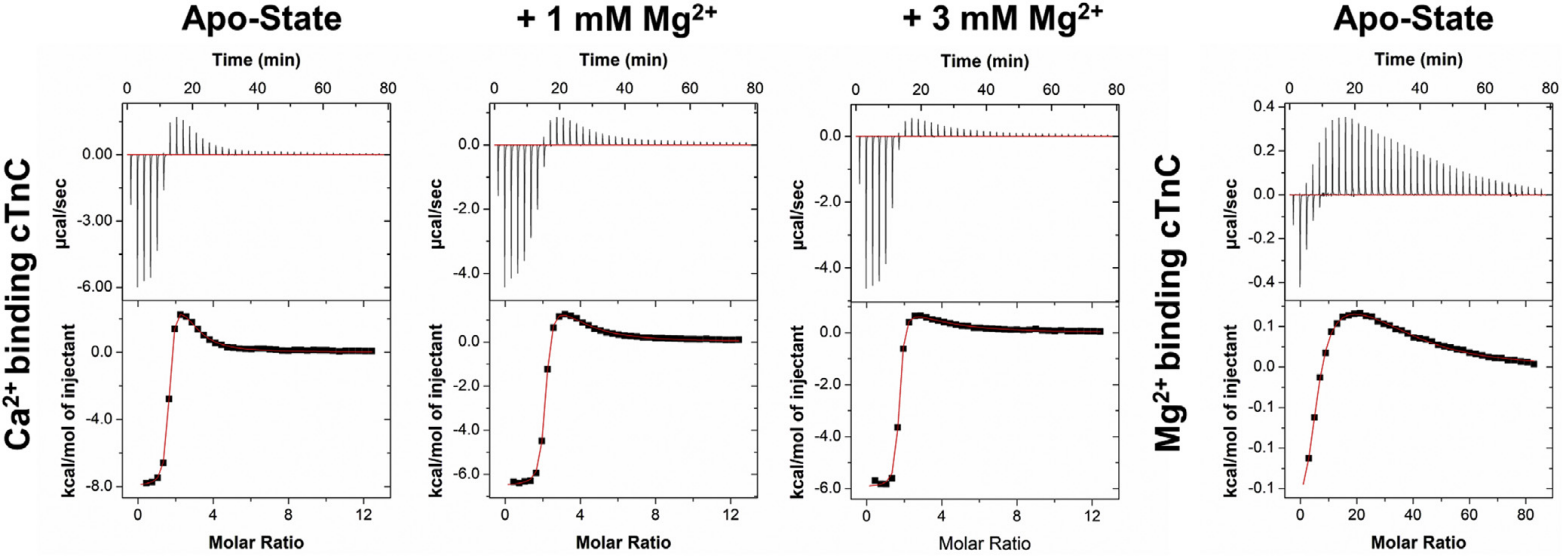
**Representative isotherms for binding of Ca**^**2+**^**and Mg**^**2+**^**to full-length cTnC.** All titrations were carried out into 100 μM full-length human WT cTnC. From *left* to *right*, the panels show the titration of 6 mM Ca^2+^ into apo-state full-length cTnC, 1 mM Mg^2+^ preincubated cTnC, and 3 mM Mg^2+^ preincubated cTnC. In the *right most panel* of this figure, 40 mM Mg^2+^ was titrated into full-length cTnC. The binding of Mg^2+^ occurs at two sets of different sites as seen in the isotherm, which contains both exothermic and endothermic components. All thermograms were fit to a “two sets-of-binding sites” model using Origin 7 MicroCal2000 ITC software package. cTnC, cardiac troponin C.

Binding of Ca^2+^/Mg^2+^ to site II is characterized by an endothermic interaction as indicated by our titrations on the N-terminal domain in this and previous publications (63, 64). From this and the ITC work on full-length cTnC by others (65), we can deduce that the exothermic heat changes seen in Figure 6 result from interactions with site III/IV. The data (Fig. 6 and Table S3) show that Mg^2+^ binds to apo-state full-length cTnC at two distinct sets of sites.

**Figure 7:**
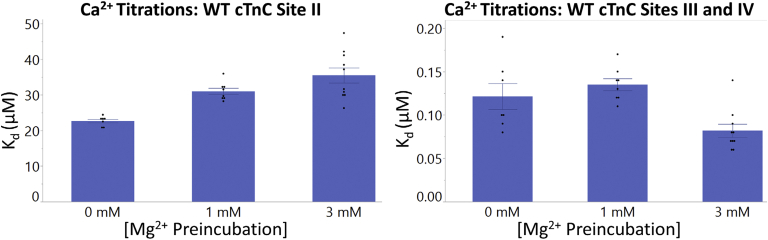
**Binding of Ca**^**2+**^**and Mg**^**2+**^**to apo-state and preincubated full-length cTnC.***Left panel*, the affinity of site II for Ca^2+^ is compared in the apo-state and with Mg^2+^ preincubation in full-length human cTnC. *Right panel*, the affinity of sites III/IV for Ca^2+^ is compared in the apo-state and with Mg^2+^ preincubation in full-length cTnC. At site II, preincubation with 1 and 3 mM Mg^2+^ caused a statistically insignificant reduction in the affinities for Ca^2+^ binding (*p* = 0.52 and *p* = 0.14). At sites III/IV, preincubation with 1 mM or 3 mM Mg^2+^ did not significantly change affinity for Ca^2+^ binding. Statistical differences were assessed through two-way ANOVA followed by Tukey's post hoc test. cTnC, cardiac troponin C.

The binding of Ca^2+^ to sites III/IV occurred with an apparent *K*_d_ of 0.12 ± 0.02 μM, characterized by an exothermic component (ΔH = −8.12 ± 0.07 kcal ∗ mol^−1^) with a positive change in entropy (T ∗ ΔS = 1.24 ± 0.07 kcal ∗ mol^−1^). In the same full-length construct, the *K*_d_ associated with binding of Ca^2+^ to site II was 22.7 ± 0.5 μM. This interaction had a positive ΔH (3.71 ± 0.06 kcal ∗ mol^−1^) and was entropically driven (T ∗ ΔS = 10.0 ± 0.01 kcal ∗ mol^−1^) (Figs. 6 and 7 and Table S3).

#### Mg^2+^ binding to apo-state full-length cTnC

Mg^2+^ binding to site II (*K*_d_ = 406.1 ± 7.9 μM) and sites III/IV (*K*_d_ = 16.7 ± 0.7 μM) was characterized by a positive ΔH (0.091 ± 0.001 kcal ∗ mol^−1^) and negative ΔH (−0.23 ± 0.01 kcal ∗ mol^−1^), respectively (Fig. 6 and Table S3). The difference in *K*_d_ values indicates that greater amounts of Mg^2+^ binding occur at the C-terminal sites, in comparison to the N-terminal site of cTnC. The interaction of Mg^2+^ with sites III/IV is two orders of magnitude weaker (*p* < 0.0001) than that seen for Ca^2+^. The interaction of Mg^2+^ with site II and sites III/IV was both entropically favorable (T ∗ ΔS = 4.71 ± 0.01 kcal ∗ mol^−1^ and T ∗ ΔS = 6.28 ± 0.03 kcal ∗ mol^−1^, respectively) and resulted in spontaneous interactions (ΔG = −4.62 ± 0.11 kcal ∗ mol^−1^ and ΔG = −6.51 ± 0.31 kcal ∗ mol^−1^, respectively).

#### Ca^2+^ binding to Mg^2+^-preincubated full-length cTnC

At greater concentrations, Mg^2+^ occupied a greater proportion of binding sites and limited binding Ca^2+^ to cTnC at all sites (Figs. 6 and 7 and Table S3). Binding of Ca^2+^ to site II was reduced by preincubation with 1 and 3 mM Mg^2+^ as indicated by an increase in *K*_d_; however, these changes were not found to be statistically significant (*p* = 0.52 and *p* = 0.14), moreover ΔH was lowered (*p* < 0.0001 for both). Binding of Ca^2+^ to sites III/IV in the presence of 1 mM Mg^2+^ resulted in a *K*_d_ (0.14 ± 0.01 μM) that was not significantly different (*p* < 0.05) than seen for the 3 mM Mg^2+^ preincubation (*K*_d_ = 0.08 ± 0.01 μM) (Fig. 6 and Table S3).

At sites III/IV, for the 1 mM Mg^2+^ preincubation, the interaction proceeded with favorable enthalpy (ΔH = −6.87 ± 0.09 kcal ∗ mol^−1^) and entropy (T ∗ ΔS = 2.50 ± 0.10 kcal ∗ mol^−1^). For the 3 mM Mg^2+^ condition, the reaction was again exothermic (ΔH = −6.19 ± 0.06 kcal ∗ mol^−1^) with a positive change in T ∗ ΔS (3.50 ± 0.06 kcal ∗ mol^−1^).

## Discussion

This study provides novel information regarding the thermodynamics that underlie the interaction between cTnC and two physiologically prevalent divalent cations. Our results significantly advance the understanding of the mechanisms and role of modifications in cellular Mg^2+^ in control of the cTnC–Ca^2+^ switch. Cellular-free Mg^2+^ is known to change in pathological conditions in the heart (66), but the mechanisms of its effects on activation of the myofilaments remain incompletely understood.

As seen in previous reports, we found the binding of Ca^2+^ to N-cTnC to be driven by entropy and unfavorable enthalpy (Table S2) (64, 65). The favorable ΔS may be due in part to the hydration enthalpy of Ca^2+^, which is thought to be on the order of ∼375 kcal ∗ mol^−1^ and slightly lower than that of Mg^2+^ (∼460 kcal ∗ mol^−1^) (67). It is also possible that the endothermic nature of these interactions results from other factors such as the exchange of protons that are transferred from the ligand to the buffer upon Ca^2+^ binding (61).

Measurement of Ca^2+^ binding to cTnC is often achieved indirectly by monitoring the fluorescence change and correlating this to the conformational change that results from the interaction. Fluorescent molecules such as 2-[4'-(iodoacetamido)anilino]naphthalene-6-sulfonic acid (59, 60, 68, 69) or reporters such as F27W (51, 70) can be used to quantify this binding interaction. At 21 °C, bovine F27W cTnC had a *K*_d_ of ∼5 μM, and 2-[4'-(iodoacetamido)anilino]naphthalene-6-sulfonic acid-labeled C35S cTnC had a *K*_d_ of ∼7 μM (20, 71). Through fluorescence-based measurement, the *K*_d_ of N-cTnC for Ca^2+^ was previously reported to be between 11.3 and 12.3 μM (70, 72). These parameters agree with our measured Ca^2+^ binding to apo-state N-cTnC and apo-state cTnC (Table S2), deviating only slightly, likely because of, different buffer and temperature conditions.

Normally, cytosolic [Mg^2+^]_free_ is maintained around ∼0.5 to 1 mM (73). At these concentrations, Mg^2+^ is known to compete with Ca^2+^ for sites III and IV. Circular dichroism has been used to show that Ca^2+^ binding to sites III/IV increases the α-helical content of cTnC, from 19 to 41% (27, 74) and causes conformational changes that remove nonpolar amino acids from the solvent-exposed environment (19). This contrasts with NMR-based visualization of N-cTnC, in which the apo-state and Ca^2+^-bound forms showed minimal structural deviation (30).

Ca^2+^ has many times greater polarizability than Mg^2+^ and lower hydration energy (75). The bare ion radius of Mg^2+^ is smaller than Ca^2+^ (0.65 *versus* 0.99 Å) (76); conversely, in its hydrated form, Mg^2+^ is larger than Ca^2+^ (4.3 *versus* 4.1 Å) (77). In other Ca^2+^-binding proteins such as calmodulin (CaM), metals with similar ionic radii are able to substitute for this cation (78, 79). Mg^2+^ is able to bind to CaM but does not induce the conformational change associated with Ca^2+^ binding; a phenomenon that is commonly observed in cell biology and is expected in cTnC (80, 81).

Normally, six oxygen atoms arranged in an octahedral geometry are thought to coordinate Mg^2+^ (82). This is one less oxygen than needed to coordinate Ca^2+^ through a pentagonal bipyramid (83). However, Ca^2+^ can be coordinated by six to eight coordinating residues (but also by as many as 12) at a distance that can vary greatly (2.3–2.7 Å) compared with a much smaller variance for Mg^2+^ coordination (2.0–2.2 Å) (84).

Ca^2+^ and Mg^2+^ are most often coordinated by oxygen atoms, and this is usually accomplished by a hydroxyl group for Mg^2+^ and a carboxyl group for Ca^2+^ (85). Ca^2+^ is most frequently coordinated by side chains of aspartic acid, glutamic acid, asparagine, followed by serine/threonine, whereas Mg^2+^ is most frequently coordinated by aspartic acid, glutamic acid, histidine, threonine, serine, or asparagine (86). EF-hand–containing proteins have also been shown to bind Mg^2+^ when there are appropriately placed negatively charged amino residues (especially in the +z and −z positions) (54, 87, 88). In site II of mammalian cTnC, there is a polar serine at the +z position (residue 69) and a negatively charged glutamic acid at the −z position (residue 76) (Fig. S1).

Data from earlier studies suggested that Mg^2+^ binds exclusively at sites III and IV of TnC (4). Shortly thereafter, a limited series of equilibrium dialysis experiments did not show competition between Mg^2+^ and Ca^2+^ for the N-terminal sites of cTnC; instead, other binding sites were suggested (89). Later still, enthalpic titrations were unable to visualize a discernable change in Mg^2+^ binding to the low-affinity sites of sTnC (50, 90). However, assuming competitive binding, fluorescence assays at room temperature determined the *K*_d_ associated with Mg^2+^ binding to be about 4 mM (91). Moreover, Ca^2+^ sensitivity of the actomyosin ATPase and force production of skinned rat cardiac cells were unaltered when Mg^2+^ was increased from 1 to 8 mM (92). However, these findings were brought into question by studies that utilized metallochromic indicators to deduce sufficiently high Mg^2+^ affinity at the regulatory sites of sTnC (42).

The observation of Mg^2+^ binding to the low-affinity site of N-cTnC has led to the suggestion that differences in affinity may be due, at least in part, to Ca^2+^ buffering, and thus, the free concentration of the ion in these experiments. Given the kinetic rates associated with these interactions, it is difficult to have confidence in EGTA-determined rates of binding (93). Moreover, the temperature sensitivity of cTnC alone can alter experimental outcomes by orders of magnitude (53, 94). Change in sensitivity in the face of altered temperature has been suggested to result mostly from binding to the low-affinity sites and possibly through interactions with other members of the cTn complex (95, 96, 97).

Experiments testing the effects of alterations in free Mg^2+^ on Ca^2+^ activation of isolated myofibrils and skinned fiber bundles from different laboratories provide corroborative findings supporting the credibility of our postulate of a role for cytosolic Mg^2+^ as a controller of cTnC function at the N lobe. Fabiato and Fabiato (98) showed that increasing the concentration of free Mg^2+^ decreases myofilament Ca^2+^ sensitivity of skinned cardiomyocytes. [Mg^2+^] affects the Ca^2+^ sensitivity of the myofibrillar ATPase as well as actomyosin tension development in both skeletal and cardiac muscle preparations (10, 39, 44, 99, 100, 101, 102, 103).

Mg^2^^+^ affinity of sites III/IV alone is not sufficient to fully explain the change in the force–negative log of Ca^2+^ concentration relationship caused by Mg^2+^ in skinned skeletal muscle fibers (104). In rabbit fast skeletal muscle, Mg^2+^ competes with Ca^2+^ for low-affinity binding sites of TnC, where it binds with an affinity of 1.9 ∗ 10^2^ M^−1^ (much lower than the 6.2 ∗ 10^6^ M^−1^ seen for Ca^2+^). The *K*_A_ associated with sites III and IV was measured to be 1.2 ∗ 10^6^ M^−1^ for Ca^2+^ and 1.1 ∗ 10^2^ M^−1^ for Mg^2+^ in canine ventricular skinned myocytes (105).

In isolated cTnC, Mg^2+^ was found to interact with site II with an apparent binding constant of 5.2 ∗ 10^2^ M^−1^. This was only slightly lower than the constant associated with Mg^2+^ binding to sites III/IV (∼10^3^ M^−1^), Ca^2+^ binding to sites III/IV (∼10^6^ M^−1^), and Ca^2+^ binding to site II (∼10^4^ M^−1^) (42).

Fluorescent probes were used to measure the Mg^2+^ affinity of site II at 15 °C (∼1.2–1.9 mM) (20). In the presence of 3 mM Mg^2+^, the *K*_d_ associated with binding of Ca^2+^ to site II of full-length cTnC was increased from 7 μM in the apo-state to 24 μM (20). Moreover, a system containing cTnC–cTnI had 2.5-fold lower Ca^2+^ affinity in the presence of 3 mM Mg^2+^ (106). Given these affinities, Tikunova and Davis (20) hypothesized that site II would be 33 to 44% saturated by 1 mM cytosolic Mg^2+^ at diastolic Ca^2+^ concentrations.

In a recent ITC study, the Mg^2+^-binding affinity of site II in lobster TnC isoforms, which are similar in sequence to human variants, was explored. Mg^2+^ affinity of site II was a single order of magnitude lower than that of Ca^2+^, such that the cations would compete for binding under physiological conditions (62).

In our experiments on N-cTnC and full-length cTnC, site II-binding affinity of Mg^2+^ was an order of magnitude lower than seen for Ca^2+^ (Fig. 2 and 7 and Tables S2 and S3). At these affinities and given the relatively high cytosolic [Mg^2+^]_free_ (79, 82), this cation would compete for binding to site II of cTnC (107). Competition experiments were also in agreement (Figs. 1 and 2) as were experiments that utilized a double mutant removing coordinating residues in site II (Figs. 4 and 5). Competition experiments were also analyzed using the model available on Origin, and the thermodynamic parameters obtained from this alternative method were comparable and within the range of errors obtained Fig. 3 and Table S2).

In order to further validate the ITC data, we also performed TI to calculate absolute binding affinities computationally. We performed these calculations for both Ca^2+^ and Mg^2+^ binding separately for both WT N-cTnC and D67A/D73A N-cTnC. For both sets of simulations, the structure of Ca^2+^-bound N-cTnC (Protein Data Bank [PDB]: 1AP4) was used as the starting parameter and restrained throughout the simulation. ITC measures the thermodynamically quantifiable closed-to-open transition of the N-cTnC molecule. TI does not allow for such a transition, rather, it quantifies only the binding interaction. In the future, the closed structure of N-cTnC (PDB: 1SPY) can be simulated to quantify the presumably lower affinity it has for each of Ca^2+^ and Mg^2+^. The difference between these sets of simulations could then be used to better corroborate the ITC data.

For Ca^2+^ binding, our TI results agreed very well with the binding affinities from ITC. For Mg^2+^ binding, the calculated absolute binding affinities were consistently underestimated by about 4 kcal ∗ mol^−1^ but showed the same relative trends. Mg^2+^ was calculated to bind more weakly than Ca^2+^ in the WT and D67A/D73A mutant, in agreement with the ITC results. The Mg^2+^ absolute binding affinities were likely underestimated for multiple reasons. First, the crystal structure of WT N-cTnC (1AP4) was bound by Ca^2+^, and no structure of Mg^2+^-bound WT N-cTnC is available. We attempted to correct for this issue by minimizing the structure with Mg^2+^ bound WT N-cTnC. Because of the lack of an exact starting structure and restraints chosen, there is still likely some error. In addition, while we did try to choose the most accurate Mg^2+^ parameters for binding affinity calculations, there are well-documented difficulties in free energy calculations for Mg^2+^, most notably that the free energy of solvation (ΔG_solvation_) is consistently underestimated (108, 109). Even when using the same Mg^2+^ force field, solvation ΔG values are also known to have large variations for Mg^2+^ depending on the exact simulation parameters used. For example, both Panteva *et al.* (109) and Li *et al.* tried to reproduce Mg^2+^ salvation-free energy using the same parameters as Åqvist but saw variations on the order of 20 kcal ∗ mol^−1^ (110). While this may be an extreme example, it illustrates the difficulty in the calculation of free-energy changes with Mg^2+^ ions involved. Given these potential errors in TI for ΔG_solvation_ of Mg^2+^, the fact that we still see relatively good agreement with the ITC data for absolute binding affinity of Mg^2+^ helps further validate the *in vitro* results.

The experiments outlined previously were designed with the intent to test the hypothesis that both Ca^2+^ and Mg^2+^ interact with all the functional EF-hand motifs in cTnC. The interaction with sites III/IV has been established for some time (91), but site II may also bind Mg^2+^. Interestingly, a hypothesis that is reconcilable with our own was initially put forth; that of six binding sites (4). In this scenario, there were two Ca^2+^-specific sites, 2 Mg^2+^-specific sites, and two sites that can bind both cations. During these experiments, only the absence of Mg^2+^ allowed for the binding sites in cTnC to be separated into low-affinity sites (∼10^5^ M^−1^) and high-affinity sites (∼10^7^ M^−1^) (4). That the presence of Mg^2+^ affects the affinity of Ca^2+^ binding to TnC is also evident in a more recent study in which a fluorescence protein–based Ca^2+^ sensor was utilized to show the reorientation of both N and C domains of TnC upon Mg^2+^ binding at sites III and IV (111). In addition, studies using the ATPase activity on the strong-binding myosin heads also demonstrates the opening of more TF active sites upon Mg^2+^ binding to C-terminal domain, supporting the notion that Mg^2+^ binding causes structural changes in TnC (112).

Binding of Mg^2+^ to site II is not expected to induce significant structural changes in N-cTnC based on previous molecular dynamics simulation data (30, 61, 64). Therefore, it is likely that the favorable ΔS associated with the interaction is due to increased degrees of freedom for water molecules that would result when stabilizing hydrogen bonds are transferred from the positively charged metal cation and the negatively charged amino acid side chains in the binding site II to the buffered environment (61).

Given that the binding of Ca^2+^ to site II of cTnC at systolic Ca^2+^ levels (0.5–1.2 μM) strengthens the interaction with cTnI and the rest of the cTn complex and the orders of magnitude differ between binding affinity at varying levels of filament complexity (4, 60, 72, 113), care must be taken when translating observations at the level of cTnC to more complex systems. We suggest that other proteins in the TF, particularly cTnI, may play a central role in the mechanism discussed here. Moreover, a further limitation may be highlighted in our approach, concerning the double mutant D67A/D73A. This mutation was able to reduce the binding of both Ca^2+^ (11.9-fold) and Mg^2+^ (1.8-fold) to site II of N-cTnC; however, the impact on binding might be expected to be greater. It is possible that the effect of this double mutant is to reduce the binding of these cations, especially Mg^2+^ through allosteric interactions. In CaM, mutation of Ca^2+^ coordinating residues within the EF-hand can have structural consequences leading to altered binding kinetics (114); this is conceivable in our double mutant. Similarly, it is possible that the competition observed between Ca^2+^ and Mg^2+^ for binding to site II of cTnC occurs through structural perturbations, which follow binding of Mg^2+^ to an allosteric site. Exploration of these limitations in future studies may shed light on the true nature of these interactions.

Our ITC results strongly suggest that Mg^2+^ binds to site III/IV and competes with Ca^2+^ for binding to site II. The amount of Mg^2+^ that binds the regulatory site II is likely to be highly dependent on the technique, biological system, and buffer conditions. In N-cTnC, occupation of site II by Mg^2+^ was again seen to reduce the amount of Ca^2+^, which was able to bind this protein, at concentrations that may have physiologically relevant consequences under normal conditions and even more so in the face of diseases that alter the Ca^2+^ sensitivity of contraction.

Moreover, increases in cAMP in the cell through α- and β-adrenergic stimulation elicit extrusion of Mg^2+^ from the cell in mammalian tissues (115, 116, 117) including cardiomyocytes (118, 119). If shown in the heart, both Na^+^-dependent and Na^+^-independent removal of Mg^2+^ from the cytosol under stressful conditions would lower cytosolic presence of this cation. Despite this, free Mg^2+^ does not fluctuate greatly under such stimulation, suggesting that buffered Mg^2+^ is removed from the cell (120). Nevertheless, this altered Mg^2+^ pool may affect the subset of ions available to compete with Ca^2+^ for binding to troponin.

Based on our binding experiments and given the previous studies cited herein, Mg^2+^ may also compete with Ca^2+^ in binding to the regulatory site II. Free Ca^2+^ is tightly regulated at rest (∼0.1 μM) despite relatively high total cytosolic concentrations (2.1–2.6 mM) (84). Mg^2+^ is also abundant in the cell but is less tightly controlled. Binding of both Ca^2+^ and Mg^2+^ to site II is endothermic and thus driven by entropy. Relative to Ca^2+^, Mg^2+^ binds site II with lower affinity; however, at physiological concentrations or with elevation of free Mg^2+^, which accompanies states of energy depletion, it may reduce Ca^2+^ binding, leading to structural perturbations that modify the contractile function of the myofilament. Conversely, Mg^2+^ can be altered by diseased states such as secondary hyperparathyroidism, which results in hypomagnesia and could potentially impact cardiac contractility (121).

## Conclusions

Our study provides insights regarding the thermodynamics of metal cation binding to cTnC. The interaction of Ca^2+^ and Mg^2+^ with cTnC is characterized by differences consistent with dissimilar ionic radius, number of required coordinating residues, as well as the energic cost of exposing hydrophobic amino acids to an aqueous environment. In the cell, these differences are functionally necessitated by dissimilar free cytosolic concentrations of each cation. Cellular Mg^2+^ is not necessarily prevalent enough to directly regulate contraction and is not thought to cause a conformational change upon binding to cTnC. However, given the affinities we have observed, its occupation of the binding site may restrict Ca^2+^ binding, disable key interactions with components of the cTn complex, such as cTnI, and prevent the subsequent conformational changes necessary for rigor-state formation. This competition for binding likely favors Ca^2+^ and is well tolerated; however, elevation of free Mg^2+^, which may accompany states of ATP depletion, for example, during ischemic stress, could have relatively significant functional consequence for cardiac force production.

## Experimental procedures

### Construct preparation and protein expression

The human *TNNC1* gene (Uniprot ID: P63316) had previously been cloned into pET21a(+) vector and had a stop codon inserted at residue 90 to create the human N-cTnC construct using the Phusion site-directed mutagenesis protocol (Thermo Scientific). This construct was transformed into the BL21(DE3) *Escherichia coli* expression strain. The double-mutant D76A/D73A construct was made using site-directed mutagenesis carried out by GenScript. Expression and purification of all constructs were carried out as described previously (63, 64). In brief, 100 ml of lysogeny broth was supplemented with 50 μg/ml ampicillin and a glycerol stock stab and grown overnight at a shaking speed of 250 rpm and 37 °C. The next day, the overnight grown culture was used to inoculate each liter of lysogeny broth with 1:100 back dilution supplemented with 50 μg/ml ampicillin. Cell cultures were grown for ∼3 h until an absorbance reached to 0.8 to 1.0 at 600 nm followed by induction with 1 mM IPTG. After 3 h, the cells were harvested by centrifugation at 6000*g* for 6 min, and the collected cell pellets were stored at −80 °C.

### Protein purification

The cell pellet was thawed and suspended in buffer A (50 mM Tris–HCl at pH 8.0 and 5 mM EDTA) and sonicated on ice at 50% amplitude with 30 s on and 30 s off for 5 min, or until there was no visible viscosity of the lysate solution. After sonication, the lysate was centrifuged at 30,000*g* for 15 min at 4 °C, and the supernatant was obtained. This centrifugation process was performed twice to ensure the removal of all cell debris before loading onto a fast-flow (FF) Q-Sepharose column (GE Healthcare). The FF Q-Sepharose column was connected with an AKTA FPLC system (GE Healthcare) and pre-equilibrated with buffer A with the addition of 1 mM DTT. After applying the clear supernatant onto the column, the solution was run at 5 ml/min with a gradual gradient mixing with buffer A and buffer B (buffer A with 0.5 M NaCl), starting from 0% buffer B up to 100% buffer B by the end of the run. Following analysis by 12% SDS-PAGE, the fractions containing purified N-cTnC were pooled and concentrated using Amicon ultracentrifugal filter device (Millipore) with a 3-kDa molecular weight cutoff.

The full-length TnC was purified using the same protocol described previously, with the addition of 30% ammonium sulphate precipitation following the sonication step. After the addition of 30% ammonium sulphate, the solution was stirred on ice for 30 min and subsequently centrifuged at 28,900*g* for 30 min at 4 °C. The supernatant was obtained and dialyzed overnight against 4 l of buffer C (50 mM Tris–HCl at pH 8.0 and 100 mM NaCl).

After purification using the FF Q-Sepharose column, the fractions containing partially pure cTnC were concentrated to 3 ml using an Amicon ultracentrifugal filter device (Millipore) with a 10-kDa molecular weight cutoff. The concentrated protein sample was further purified by a HiPrep 26/60 Sephacryl S-100 column size-exclusion chromatography (GE Healthcare), which was equilibrated with buffer C. After confirming the purity of the protein on a 12% SDS-PAGE gel, all fractions containing the purified cTnC were combined, aliquoted, and stored at −80 °C prior to pre-ITC dialysis.

### Protein dialysis

To generate the apo-state protein, TnC was first dialyzed against 2 l of 50 mM Hepes at pH 7.2, 150 mM KCl, 2 mM EDTA, and 15 mM β-mercaptoethanol, followed by another dialysis against the same buffer with no EDTA added. Each of these dialysis steps was completed at 4 °C for a minimum of 4 h. A third dialysis was performed for a minimum of 16 h overnight against 2 l of 50 mM Hepes at pH 7.2, 150 mM KCl, and 2 mM β-mercaptoethanol. An extinction coefficient of 1490 and 4595 M^−1^ cm^−1^ and a molecular weight of 10.1 and 18.4 kDa was used to determine protein concentration for the N-cTnC and full-length cTnC constructs, respectively, by 280 nm UV–visible spectroscopy using a NanoDrop 2000 spectrophotometer (Thermo Scientific). The final dialysis buffer was used to dilute the protein samples to a final concentration of 200 μM for the N-terminal construct and 100 μM for full-length cTnC as described previously (64).

Standard 1.0 M CaCl_2_ and MgCl_2_ stock solutions (Sigma) were serially diluted in the final dialysis buffer to produce 6 mM Ca^2+^ and 40 mM Mg^2+^, respectively. The same standards were used to produce 4 mM Ca^2+^ and 20 mM Mg^2+^ titrants for the N-cTnC experiments. Given the key role of protein concentration in determination of affinity, we aimed to ensure consistency and did so through dilution of the protein from stock solutions with care taken to minimize human and pipetting error to fall at the minimum possible using recently calibrated instrumentation.

### Isothermal titration calorimetry

The ITC experiments were carried out in a MicroCal ITC200 instrument (Malvern). Repeat titrations were used to ensure reproducibility. The sample cell was set at 25 °C, 200 μl of the protein was loaded, and the experiment was carried out at the same temperature. For the N-cTnC, 19 injections of the titrant were used with the first being a dummy injection of 0.8 μl and the subsequent 18 injections, 2 μl each. For these experiments, 4 mM Ca^2+^ was titrated into 200 μl of 200 μM apo-state N-cTnC as the baseline condition. For the full-length cTnC, 6 mM Ca^2+^ was titrated into 200 μl of 100 μM apo-state full-length cTnC with a dummy injection of 0.5 μl and 38 injections of 1 μl. The time interval between injections was 120 s, and stirring speed was set at 1000 rpm throughout each experiment.

### Analysis of results

Titration data were analyzed using MicroCal2000 for ITC through Origin 8.0 (OriginLab). Raw heats were integrated and fit by a least-squares algorithm using a “single-binding site” model for the N-cTnC titrations and a “two sets-of-binding sites” model for the full-length cTnC titrations to calculate the thermodynamic parameters. As a point of comparison, the “competition” model on Origin was also used to study the binding of Ca^2+^ and Mg^2+^ to the apo-state N-cTnC as well as in the presence of 1 mM of the counter ion (Fig. 3) (122). For the N-cTnC constructs (apart from the Ca^2+^ into apo-state N-cTnC condition), the N (number of binding sites) associated with each interaction was necessarily constrained to equal 1.00 to facilitate curve fitting without altering protein concentration. The baseline condition was repeated daily, and the consistency of the thermodynamic parameters in these sets of titrations indicates protein quality and function throughout the set of experiments. If multiple ligands were simultaneously present in the reaction mixture, an “apparent affinity” was determined for the injected titrant.

JMP 14.0 software package was used for statistical analysis. ANOVA was used to identify differences in each studied thermodynamic parameter from the N-cTnC and full-length cTnC titrations: in the apo-state and competition experiments, including both the WT and double-mutant proteins (N-cTnC only). Tukey's post hoc test was subsequently used to explore where the differences lie with *p* < 0.05 considered the threshold for statistical significance.

### Thermodynamic integration

Starting from the representative model of PDB: 1AP4 (30), which contains N-cTnC with a single Ca^2+^ ion bound, the system was solvated with a 12 Å-padded transferable intermolecular potential 3P water box and neutralized with Na^+^ in Amber16 (123). The system was also prepared similarly for only the Ca^2+^ ion in a 12 Å-padded transferable intermolecular potential 3P water box. The alchemical thermodynamic cycle used for ligand binding was described in detail previously (124). In short, TI was performed using the following three steps for Ca^2+^ in protein: turn on restraints, turn off charge, and turn off van der Waals forces. The specific distance restraints used in all systems can be found in Table S1. In addition, TI was performed for the following two steps for Ca^2+^ in water: turn off charge and turn off van der Waals forces. Each step of the thermodynamic cycle was performed with the coupling parameter (λ) ranging from 0.0 to 1.0 in increments of 0.1. For each simulation, the system was minimized (2000 cycles) and heated (0.5 ns) before the 5 ns production run at 300 K using the ff14SB force field (125). These calculations were also performed on the D67A/D73A mutated system. The mutations were imposed on the 1AP4 representative model using PyMOL (126).

For the calculation of Mg^2+^-binding affinity, Ca^2+^ was replaced with Mg^2+^ in the 1AP4 representative model since no Mg^2+^-bound N-cTnC structure was available in the PDB. In order to generate more accurate restraints and starting coordinates for the TI calculations, a minimization was performed on the structure in Amber (2000 cycles). Following the minimization, TI simulations were run similarly as for Ca^2+^. However, because of previously documented errors in the default Mg^2+^ parameters, the ΔG_solvation_-optimized Mg^2+^ parameters from Li *et al.* were used (109, 127). These calculations were also performed on the D67A/D73A mutated system.

To calculate absolute binding affinities for the ions, the change in free energy (ΔG) was calculated for each step in the thermodynamic cycle by integrating the potential energy with respect to the coupling parameter, λ (128). Two corrections were made to these calculated ΔG values. The first correction was necessary because of the introduction of the distance restraints (as described in the study by Boresch *et al.* (129)), which quantified the free energy cost of restraining the ion to the binding site. The second correction was performed to correct the charged system (as described in the study by Rocklin *et al.* (130)) to revise the free energy for the fact that the system is charged during the disappearance of the charged ions. The overall ΔG of binding was the change in free energy between the ions in complex with the protein (ion in protein steps 1, 2, and 3) and the ions in water (ion in water steps 1 and 2). For each system, five independent runs were performed, and results were averaged.

## Data availability

All data presented in this article are contained within the article.

## Supporting information

This article contains supporting information.

## Conflict of interest

The authors declare that they have no conflicts of interest with the contents of this article.
