# Supplementary material for: Binding of calcium and magnesium to human cardiac troponin C
Source: J Biol Chem. 2021 Feb 3;296:100350. doi: 10.1016/j.jbc.2021.100350 (PMC7961095; doi:10.1016/j.jbc.2021.100350)
Supplement: Supplemental Figures S1–S2 and Tables S1–S3 [file mmc1.docx]

**Supplementary Appendix**


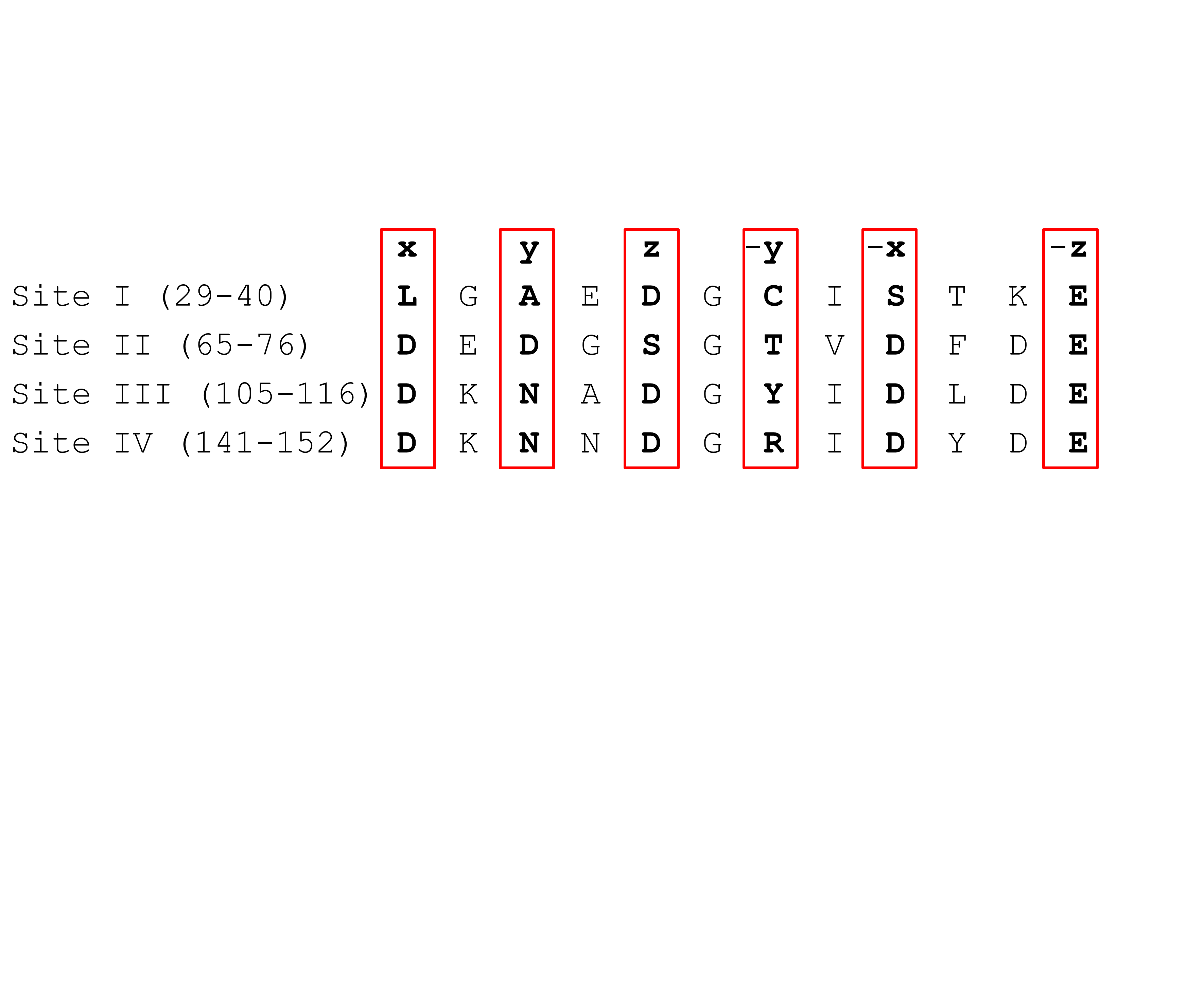


Figure S1 - Sequence alignment of the 4 EF hand binding motifs in cTnC

The coordinating residues within EF hands I-IV are shown with the residue number listed in brackets. Each of residues x, y, z, -y, -x, and -z, that make up the helices of the pentagonal bipyramid are indicated. The conservation of the amino acids in each of the coordinating residues between sites II, III, and IV is striking as is the clear differences seen in site I.


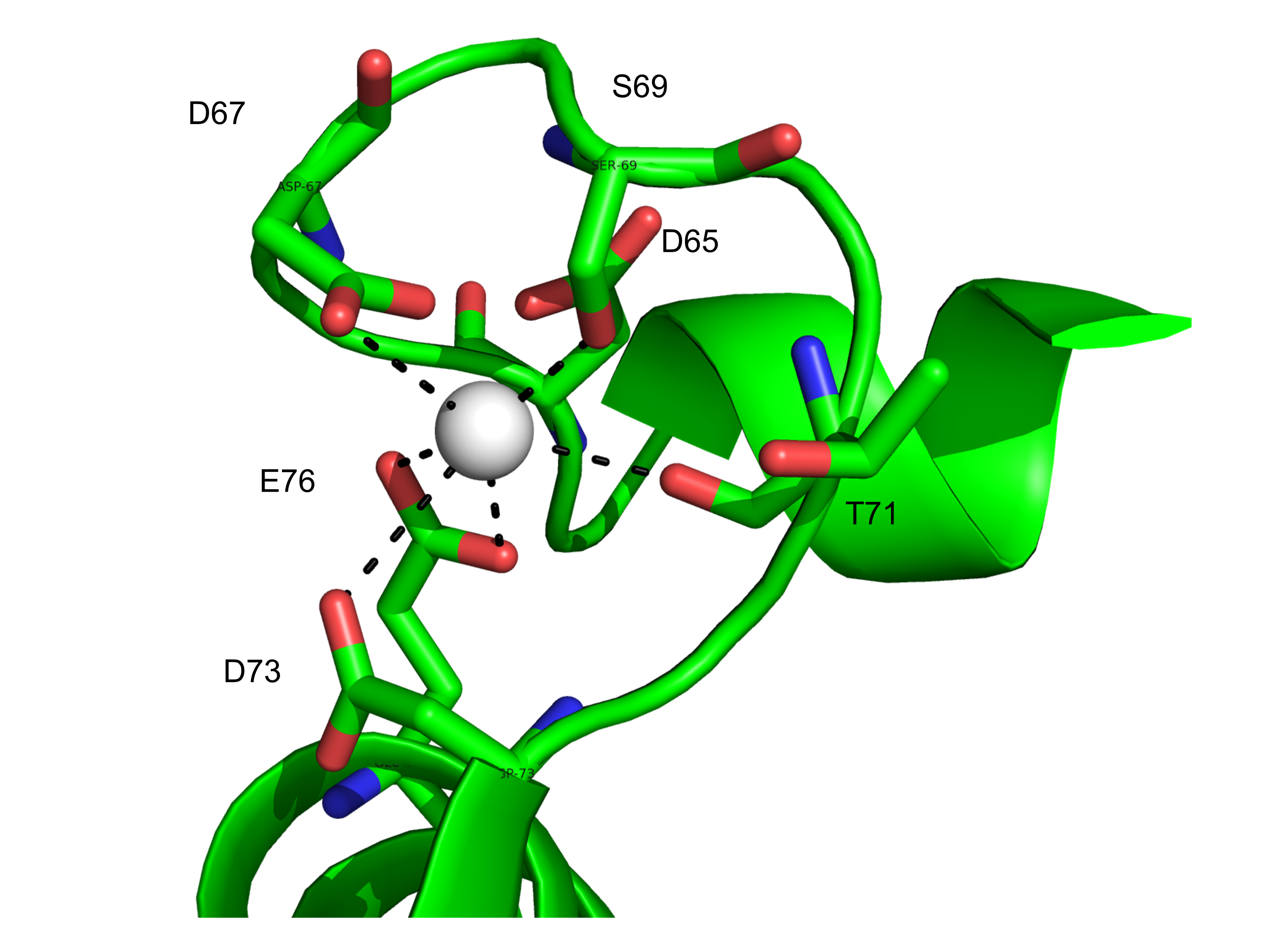


Figure S2 - Depiction of site II of WT cTnC coordinating a divalent cation

The D67A/D73A double mutant removes two of the coordinating residues within the EF hand of site II in N-cTnC. The goal of this double mutation was to compare the reduced amount of binding of Ca^2+^ and Mg^2+^ and to gain insight into the locus of binding for each cation. The residue coordinating residues at the vertices of the pentagonal bipyramid are as follows: x (D65), y (D67), z (S69), -y (T71), -x (D73), and -z (E76). The figure was generated using PyMOL and adapted from the PDB:1J1E X-ray structure.

Table S1 - Ion restraints used for thermodynamic integration


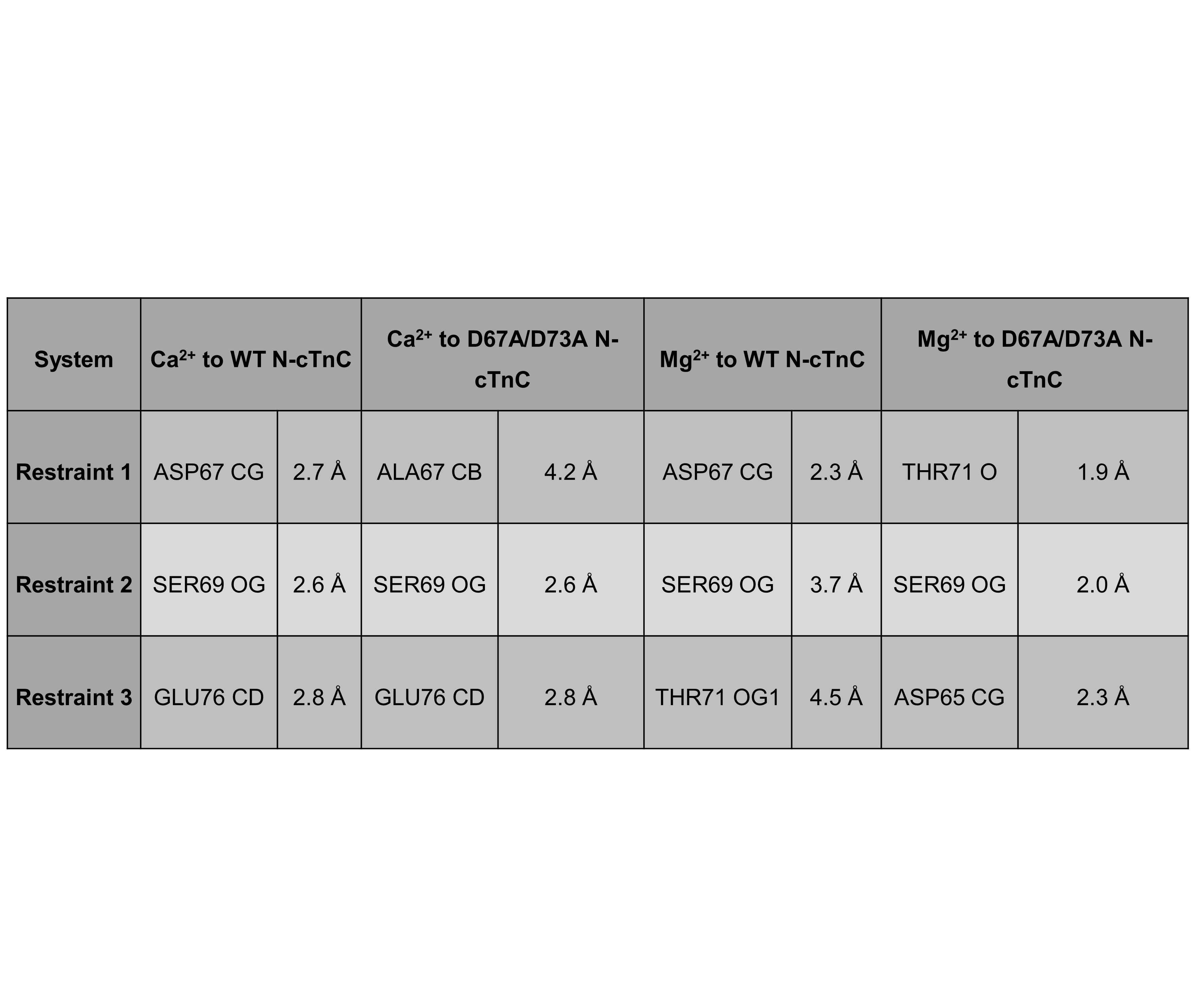


For each system, 3 restraints to the ion were used. Table shows the atom and the distance to the restrained ion.

Table S2 - Thermodynamic Parameters for Ca^2+^ and Mg^2+^ binding to N-cTnC


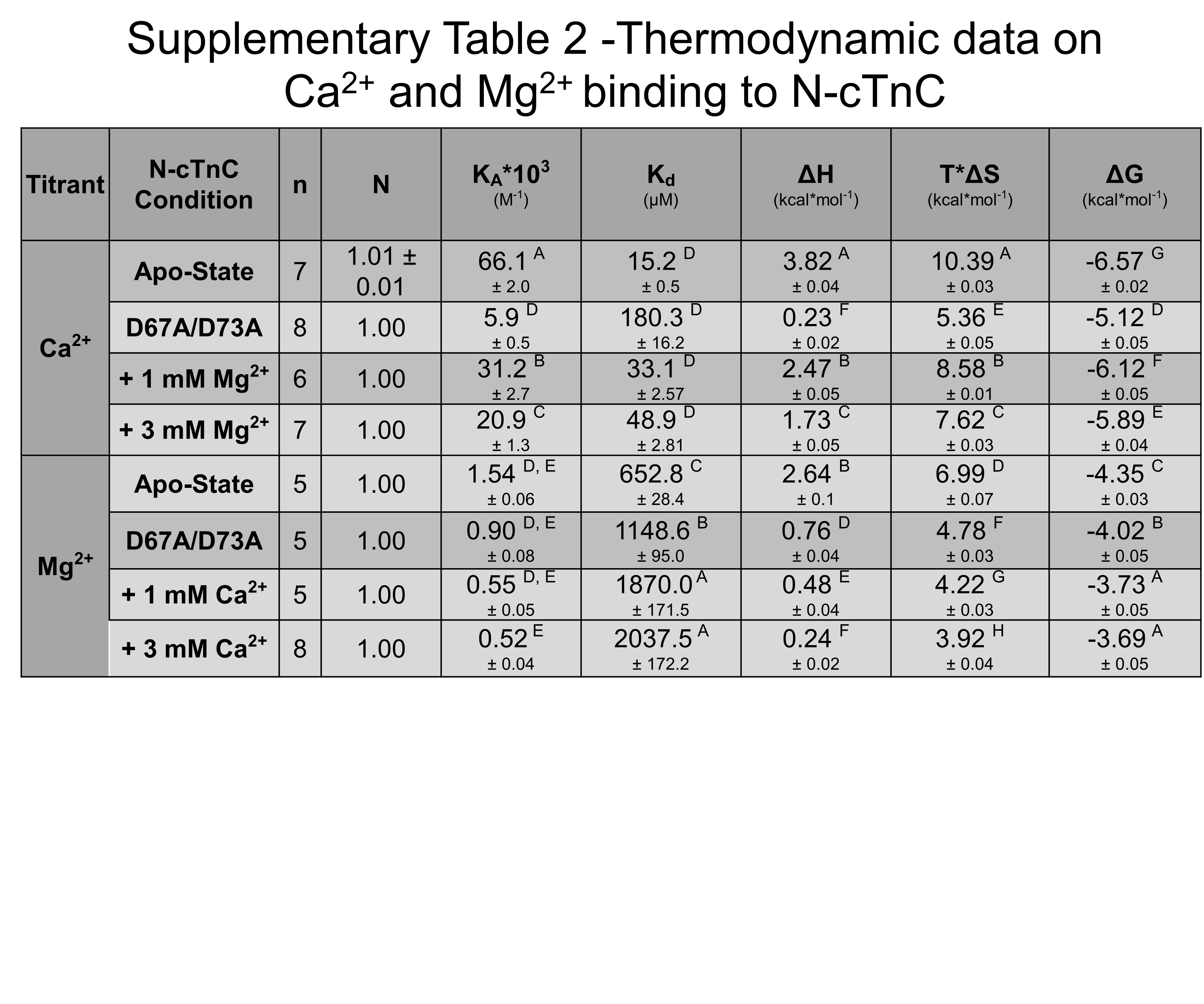


In this table, n indicates the experimental repeats and N indicates the stoichiometry number. 4 mM Ca^2+^ and 20 mM Mg^2+^ titrations into 200 µM N-cTnC were the baseline conditions. For the Ca^2+^ titrations, pre-incubation with 1 or 3 mM Mg^2+^ prior to titrations was used to study the site II occupation by both cations. The affinity and enthalpy change associated with the interaction was decreased with increasing amounts of Mg^2+^ pre-incubated. For the Mg^2+^ titrations, pre-incubation with 1 or 3 mM Ca^2+^ prior to titrations was used to study the binding of both cations to site II. Increasing the concentration of Ca^2+^ decreased the binding affinity of Mg^2+^ to site II and decreased the enthalpy change, therefore less Mg^2+^ binds N-cTnC in the presence of Ca^2+^. The D67A/D73A mutant was used to reduce the binding of both cations to the EF hand of site II, albeit to a different extent for each. All parameters are displayed as mean ± SEM, with the exception of the stoichiometric ratio for the Mg^2+^ binding which was constrained to 1.00 to facilitate fitting. For each factor, ANOVA was carried out indicating significance. Subsequently, Tukey’s post-hoc test was used to determine which conditions differed significantly (p<0.05), titrations not linked with the same superscripted letter were significantly different. The first letter of the alphabet indicates the largest mean and each subsequent letter denotes a significantly lower mean.

Table S3 - Thermodynamic Parameters for Ca^2+^ and Mg^2+^ binding to full-length cTnC


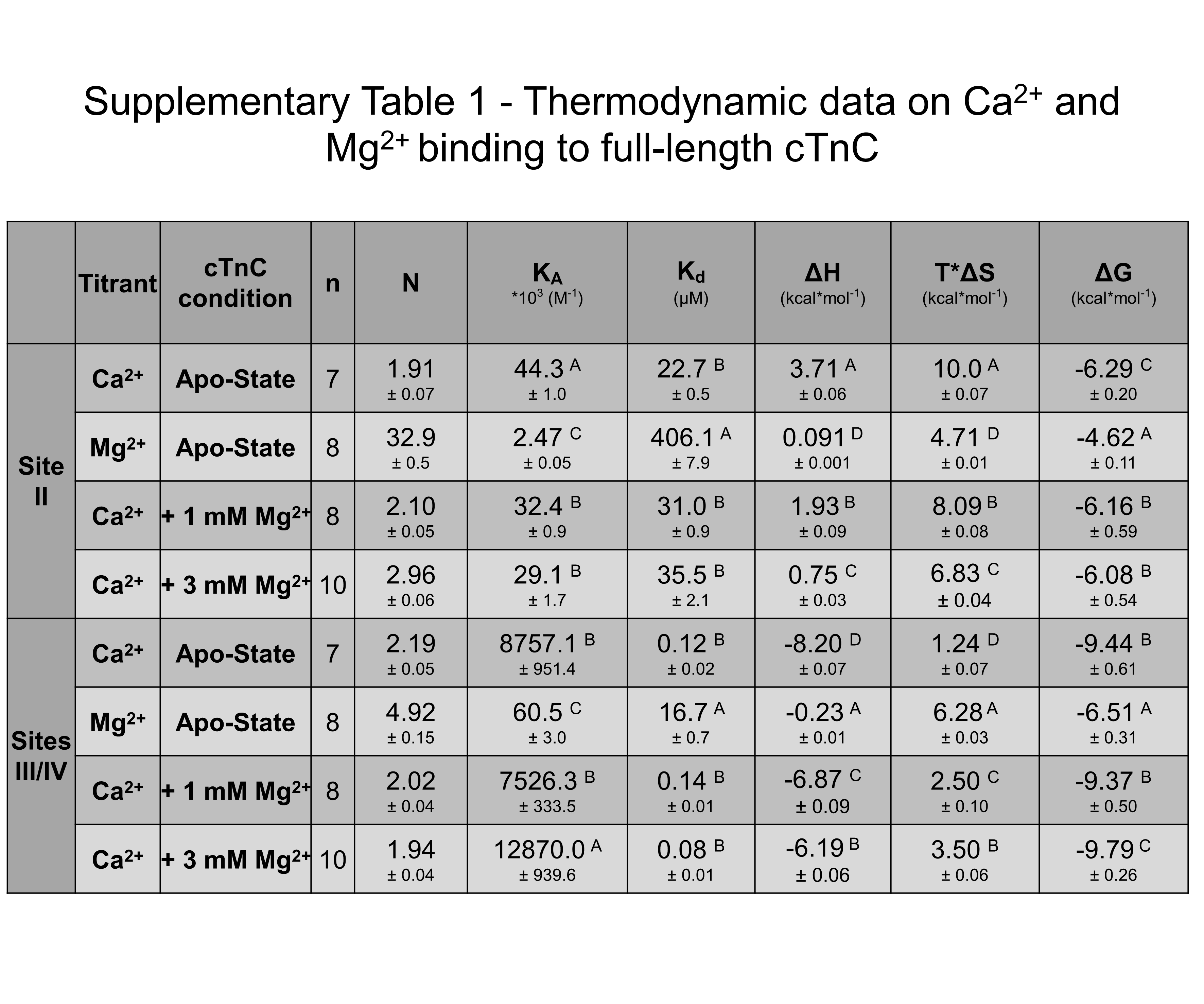


In this table, n indicates the experimental repeats and N indicates the stoichiometry number. 6 mM Ca^2+^ and 40 mM Mg^2+^ titrations into 100 µM N-cTnC were the baseline conditions. Ca^2+^ was also titrated into protein pre-incubated with 1 and 3 mM Mg^2+^ to study the competition for all three binding sites. Binding to site II was analyzed separately from binding to sites IIII/IV where analysis of variance was used to test for a significant difference in the means for each thermodynamic parameter. At the level of each parameter, following initial ANOVA, Tukey’s post hoc test was carried out. The results of this test are indicated by superscripted letters, where conditions with unique letters are significantly different (p < 0.05). The stoichiometry of Mg^2+^ binding to site II of cTnC is nonsensically high, consistent with the very low binding affinity. However, the fit of the data has been visually assessed and is on the order of the goodness of fit for the other isotherms as indicated by a comparable chi-square value. The very different K_d_ value obtained for this titration, in comparison to the others may still be expected to indicate a biologically relevant difference.
